# Association of ACAG with short-term mortality in liver failure patients: a retrospective analysis based on the MIMIC-IV database

**DOI:** 10.1038/s41598-026-39253-5

**Published:** 2026-03-21

**Authors:** Hang Wang, Shuangli Li, Shunhao Lai, Yunjian Sheng

**Affiliations:** Department of Infectious Diseases, The Affiliated Hospital of Southwest Medical University, NO.25, Taiping Street, Jiangyang District, Luzhou, 646000 Sichuan China

**Keywords:** ACAG, Liver failure, Mortality, MIMIC-IV database, Biomarkers, Diseases, Gastroenterology, Medical research, Risk factors

## Abstract

Traditional scoring systems are challenging for early diagnosis and intervention in liver failure. While albumin-corrected anion gap (ACAG) predicts adverse outcomes in various conditions, its effectiveness in liver failure remains unclear. This study analyzed hepatic failure patients from MIMIC-IV, stratified into Q1-Q4 by ACAG quartiles. Kaplan-Meier survival curves examined mortality differences. The connection of ACAG with mortality was examined using Cox regression and restricted cubic spline (RCS) analyses. Additionally, we compared the predictive performance of ACAG, MELD, and their combination for mortality using receiver operating characteristic (ROC) curve analysis, calculated the area under the curve (AUC), and compared the AUC values using the DeLong test. Finally, subgroup analyses were conducted to evaluate the relationship between ACAG and prognosis in different types of liver failure. Current study involving 2,016 patients with liver failure revealed 30- and 90-day mortality rates of 27.23% and 30.01%, respectively. Kaplan-Meier survival curves demonstrated mortality increased with ACAG levels. Multivariate Cox regression confirmed ACAG’s positive association with mortality [HR 1.032 (30-day), 1.031 (90-day); both *P* < 0.001], and the RCS showed a linear relationship. The AUCs for 30-day mortality were 0.641 (ACAG), 0.634 (MELD), and 0.668 (combined); the corresponding values for 90-day mortality were 0.630, 0.621, and 0.654. The AUC of the combined model was significantly higher than that of ACAG alone (*P* = 0.0009 and 0.0016) and MELD alone (*P* = 0.0004 and 0.0002) for 30- and 90-day mortality, respectively. Although ACAG yielded a marginally higher AUC than MELD, DeLong’s test indicated no significant difference (*P* = 0.7035 and 0.5387 for 30-and 90-day mortality, respectively). Subgroup analyses observed interactions in the age subgroups and CRRT subgroups; ACAG was consistently elevated in non-survivors across all liver-failure subtypes. Our study demonstrates that the ACAG index is significantly associated with mortality in patients with hepatic failure; its integration with the MELD score further enhances predictive accuracy.

**Supplementary Information:**

The online version contains supplementary material available at 10.1038/s41598-026-39253-5.

## Introduction

 Liver failure, a multifactorial syndrome, features jaundice, clotting issues, hepatorenal syndrome, liver-related encephalopathy, and abdominal fluid buildup, with high short-term mortality^1,2^. Consequently, it is essential to detect liver failure promptly. Liver failure therapy relies on three key principles: early detection and treatment of the cause, proactive complication management, and systematic patient evaluation. Accurate early prognosis is vital to prevent liver dysfunction progression^3–5^. Several models are currently used to assess the severity of liver failure, such as the Model for End-Stage Liver Disease Score (MELD), the Chronic Liver Failure Consortium Acute-on-Chronic Liver Failure Score (CLIF-C ACLF), the Model for End-Stage Liver Disease-Sodium Score (MELD-Na), and the Child-Turcotte-Pugh (Child-Pugh) score. However, these models rely heavily on the severity of organ failure and laboratory indicators, which often appear normal until later stages, hindering early diagnosis and intervention^6–8^. Therefore, a simple, cost-effective, and highly sensitive index is urgently needed to evaluate the prognosis of individuals experiencing liver failure.

Serum albumin primarily reflects the body’s nutritional status, hepatic and renal function, inflammation, and infection^9^. Patients with liver failure usually have hypoalbuminemia, which is more common in severely ill patients and has a strong association with higher mortality and poorer prognosis^10,11^. The anion gap (AG) is an important indicator for assessing acid-base disturbance and the disease stage, the results of which are influenced by the net negative charge carried by the albumin molecule^12^. Thus, the albumin-corrected anion gap (ACAG) more accurately reflects unmeasured anion levels and the patient’s acid-base status^13,14^. Research indicates that elevated ACAG concentrations are related to poorer health outcomes among patients in critical condition, particularly those with heart failure, sepsis, or acute pancreatitis^15–18^. While ACAG’s role in hepatic pathophysiology is recognized, its prognostic value in liver failure remains unclear. This study investigates ACAG’s correlation with clinical outcomes to assess its biomarker potential.

## Materials and methods

### Database

This retrospective investigation examined the connection of ACAG with liver failure outcomes using data from the MIMIC-IV database (v3.1). The MIMIC-IV repository contains anonymized ICU patient records sourced from Beth Israel Deaconess Medical Center (BIDMC), spanning the period 2008 to 2022^19^. The database was accessed by an author (Hang Wang) who had completed compliance certification and extracted data (certification number: 68167728). This study was exempted from ethical review because the database had anonymized patient information.

### Research population

The selection criteria for this research were established in the following manner: (1) Individuals confirmed to have liver failure were identified using ICD-10 diagnostic codes (K704, K7040, K7041, K721, K7210, K7211, K720, K7200, K7201, K729, K7290, K7291); (2) Patients were first hospitalized in ICU for any length; (3) patients were 18 years or older; (4) only patients whose first ICU admission contained serum AG and albumin test results within 24 h were included. Further screening excluded patients who met the following criteria: (1) not a first-time ICU admission; (2) younger than 18 years; (3) no serum AG and albumin tests on the first day of ICU hospitalization; (4) Patients who underwent liver transplantation during their ICU hospitalization or within the 90-day follow-up period.

### Data extraction

The clinical information we collected on patients included the following elements: (1) Basic demographic information: age, weight, sex, marital status, and race. (2) Vital signs: respiratory rate, heart rate, and oxygen saturation (SpO2). (3) Comorbidities: hypertension, chronic kidney disease (CKD), diabetes mellitus (DM), chronic obstructive pulmonary disease (COPD). (4) Laboratory parameters: hemoglobin (HB), white blood cells (WBC), platelets (PLT), red blood cells (RBC), hematocrit (HCT), red blood cell distribution width (RDW), potassium, sodium, calcium, glucose, aspartate aminotransferase (AST), alanine aminotransferase (ALT), activated partial thromboplastin time (APTT), prothrombin time (PT). (5) Clinical scores: Charlson Comorbidity Index, Sequential Organ Failure Assessment (SOFA), Glasgow Coma Scale (GCS), MELD. (6) Therapeutic methods: mechanical ventilation (MV), vasopressin (VP), and continuous renal replacement therapy (CRRT). The covariates were selected based on clinical relevance and prior studies on liver disease prognosis^14^.

### Calculation formula and primary outcomes

Standard anion gap (AG): AG (mmol/l) = (sodium + potassium) - (chloride + bicarbonate)^20^.

Albumin-corrected anion gap (ACAG): ACAG (mmol/L) = [4.4-albumin (g/dL)] *2.5 + AG^21^.

Primary outcomes: 30- and 90-day mortality. 30-day mortality reflects early (often in-hospital) events typical of acute liver failure and acute on chronic liver failure^6,7^, whereas 90-day mortality reflects outcomes such as late sepsis, re-bleeding or progressive organ failure among ICU survivors^5^. Both endpoints are standard in liver-failure research.

### Statistical analysis

 Numerical data were expressed as mean ± standard deviation (SD) (normally distributed) or median (P25-P75) (non-normally distributed). Normally distributed variables were analyzed using parametric tests (t-test/ANOVA), while nonparametric methods (Mann-Whitney U/Kruskal-Wallis) were applied to skewed distributions. Categorical data were assessed via the chi-square test, reported as frequencies (n%). Kaplan-Meier analysis with log-rank test evaluated outcome occurrence across ACAG levels. Multivariate Cox regression, adjusted for univariate-identified confounders, examined ACAG-mortality associations, presenting HRs (95% CI); multicollinearity was evaluated using variance inflation factors (VIF) (Table S3). Trend significance (P-trend) was calculated for ordered groups. RCS (restricted cubic spline) regression modeled potential ACAG-mortality nonlinearity, with knots placed at the 14th, 20th, and 29.25th percentiles. Receiver operating characteristic (ROC) curve analysis compared the predictive performance of ACAG, MELD, and their combined use. The DeLong test is used to compare AUC values. Subgroup analyses was conducted based on liver failure type, age, gender, ethnicity, hypertension, chronic lung disease, CRRT, mechanical ventilation, and vasopressor.

The analysis and process of data were conducted using DecisionLinnc v1.0.9, a versatile platform designed for integrating various programming languages. This tool facilitates data manipulation, analytical tasks, and machine learning via an intuitive visual interface. Statistical significance was defined using a P-value cutoff of 0.05.

## Result

### Baseline patient characteristics

This study included 2016 patients suffering from liver failure, with the flow chart shown in Figure S1. The median age was 61 (52–71), and 60.47% were male. Diagnoses included: acute and subacute liver failure (58.58%), chronic liver failure (1.69%), alcoholic liver failure (14.09%), and unclassified liver failure (25.64%). The 30-day mortality rate was 27.23%, rising to 30.01% at 90-days. Patients were categorized into four groups according to ACAG quartiles (Table 1). Patients with higher ACAG exhibited elevated heart and respiratory rates (*p* < 0.001), increased prevalence of diabetes (*p* = 0.002) and chronic kidney disease (*p* = 0.004), and higher SOFA (*p* < 0.001), Charlson (*p* = 0.01), and MELD scores (*p* < 0.001). Laboratory findings included lower calcium levels (*p* < 0.001) and elevated WBC, PLT, ALT, AST, potassium, glucose, PT, and APTT (all *p* < 0.001). The highest ACAG quartile had higher 30- and 90-day mortality and greater CRRT use (all *p* < 0.001).

**Table 1:** Baseline characteristics of patients with liver failure according to ACAG classification.

Variables	Overall (*N* = 2016)	Quartile 1 (*n* = 509)	Quartile 2 (*n* = 500)	Quartile 3 (*n* = 521)	Quartile 4 (*n* = 486)	*p*
**Age (year)**	61.00 (52.00–71.00)	59.00 (50.00–68.00)	62.00 (53.00–71.00)	62.00 (53.00–73.00)	62.00 (52.00–73.00)	< 0.001
**Weight (kg)**	83.00 (68.80–98.80)	84.90 (70.40–100.00)	81.47 (68.20–97.85)	81.50 (66.70–97.40)	84.01 (69.70–100.00)	0.074
**Gender**,** n(%)**						0.68
**Female**	797.00 (39.53%)	192.00 (37.72%)	195.00 (39.00%)	209.00 (40.12%)	201.00 (41.36%)	
**Male**	1,219.00 (60.47%)	317.00 (62.28%)	305.00 (61.00%)	312.00 (59.88%)	285.00 (58.64%)	
**Marital status**,** n(%)**						0.008
**Divorced**	199.00 (9.87%)	52.00 (10.22%)	50.00 (10.00%)	36.00 (6.91%)	61.00 (12.55%)	
**Married**	964.00 (47.82%)	232.00 (45.58%)	263.00 (52.60%)	240.00 (46.07%)	229.00 (47.12%)	
**Single**	725.00 (35.96%)	200.00 (39.29%)	153.00 (30.60%)	207.00 (39.73%)	165.00 (33.95%)	
**Widowed**	128.00 (6.35%)	25.00 (4.91%)	34.00 (6.80%)	38.00 (7.29%)	31.00 (6.38%)	
**Race**,** n (p%)**						0.206
**Black**	197.00 (9.77%)	44.00 (8.64%)	43.00 (8.60%)	53.00 (10.17%)	57.00 (11.73%)	
**Other**	636.00 (31.55%)	148.00 (29.08%)	153.00 (30.60%)	173.00 (33.21%)	162.00 (33.33%)	
**White**	1,183.00 (58.68%)	317.00 (62.28%)	304.00 (60.80%)	295.00 (56.62%)	267.00 (54.94%)	
**Vital signs**						
**HR (beats/min)**	93.00 (80.00–109.00)	89.00 (77.00–105.00)	93.00 (79.00–107.50)	95.00 (81.00–112.00)	97.00 (83.00–111.00)	< 0.001
**RR (beats/min)**	20.00 (16.00–25.00)	19.00 (16.00–24.00)	20.00 (16.00–24.00)	20.00 (17.00–25.00)	22.00 (18.00–26.00)	< 0.001
**SpO2**	97.00 (94.00–100.00)	98.00 (95.00–100.00)	97.00 (94.00–100.00)	97.00 (94.00–100.00)	97.50 (95.00–100.00)	0.166
**Score**						
**SOFA**	10.00 (7.00–12.00)	8.00 (6.00–10.00)	8.00 (6.00–11.00)	10.00 (8.00–13.00)	12.00 (9.00–14.00)	< 0.001
**GCS**	15.00 (13.00–15.00)	15.00 (13.00–15.00)	15.00 (13.00–15.00)	15.00 (13.00–15.00)	15.00 (13.00–15.00)	0.975
**Charlson index**	6.00 (4.00–8.00)	5.00 (4.00–7.00)	6.00 (4.00–8.00)	6.00 (4.00–8.00)	6.00 (4.00–8.00)	0.01
**MELD**	18.03 (11.16–25.68)	12.86 (7.35–19.34)	15.71 (9.58–21.81)	19.83 (13.02–27.26)	25.14 (18.22–31.77)	< 0.001
**Laboratory tests**						
**RBC (K/uL)**	3.03 (2.52–3.77)	2.97 (2.49–3.72)	2.98 (2.52–3.70)	3.05 (2.50–3.80)	3.16 (2.60–3.83)	0.233
**WBC (K/uL)**	11.80 (7.55–18.00)	10.30 (6.50–14.20)	11.30 (7.30–17.45)	12.50 (7.90–18.30)	14.55 (9.30–21.10)	< 0.001
**Hemoglobin (g/L)**	9.30 (7.80–11.15)	9.20 (7.70–11.00)	9.10 (7.80–10.90)	9.30 (7.70–11.40)	9.60 (8.00–11.20)	0.383
**Platelets (K/uL)**	125.00 (71.00–198.00)	107.00 (64.00–173.00)	123.00 (73.00–191.50)	128.00 (69.00–203.00)	143.00 (78.00–221.00)	< 0.001
**Hematocrit (%)**	28.60 (24.00–34.60)	28.20 (24.00–34.40)	28.20 (24.10–33.10)	29.00 (23.80–35.00)	29.85 (24.60–35.30)	0.053
**RDW (%)**	16.70 (14.70–19.00)	16.50 (14.50–18.60)	16.70 (14.70–19.20)	16.80 (14.80–19.10)	16.65 (14.80–19.10)	0.172
**Potassium (mEq/L)**	4.30 (3.80–5.00)	4.30 (3.90–4.80)	4.20 (3.70–4.90)	4.30 (3.70–5.00)	4.60 (3.90–5.30)	< 0.001
**Sodium (mEq/L)**	137.00 (133.00–141.00)	137.00 (133.00–141.00)	137.00 (133.00–141.00)	137.00 (133.00–140.00)	137.00 (133.00–141.00)	0.681
**Calcium(mEq/L)**	8.30 (7.60–8.90)	8.30 (7.80–8.90)	8.30 (7.80–8.80)	8.30 (7.60–8.90)	8.00 (7.30–8.80)	< 0.001
**Glucose (mg/dL)**	133.00 (105.00–185.00)	129.00 (105.00–165.00)	131.00 (106.00–174.50)	133.00 (105.00–187.00)	144.00 (104.00–234.00)	< 0.001
**PT (s)**	19.50 (15.50–25.80)	18.50 (14.90–24.40)	18.50 (15.25–23.50)	19.80 (15.30–26.80)	21.90 (17.00–30.30)	< 0.001
**APTT (s)**	36.40 (30.70–46.90)	35.30 (30.10–44.70)	35.95 (30.10–44.40)	36.80 (30.70–48.60)	37.90 (31.60–50.60)	< 0.001
**ALT(IU/L)**	58.00 (25.00–297.50)	45.00 (21.00–135.00)	43.00 (22.00–146.00)	56.00 (24.00–244.00)	200.00 (46.00–865.00)	< 0.001
**AST(IU/L)**	121.00 (52.00–488.00)	82.00 (46.00–246.00)	86.00 (47.00–234.50)	122.00 (47.00–381.00)	404.50 (105.00–1,617.00)	< 0.001
**Comorbidities**						
**Hypertension**,** n (p%)**						0.952
**No**	1,507.00 (74.75%)	378.00 (74.26%)	372.00 (74.40%)	389.00 (74.66%)	368.00 (75.72%)	
**Yes**	509.00 (25.25%)	131.00 (25.74%)	128.00 (25.60%)	132.00 (25.34%)	118.00 (24.28%)	
**CKD**,** n (p%)**						0.004
**No**	1,572.00 (77.98%)	425.00 (83.50%)	389.00 (77.80%)	391.00 (75.05%)	367.00 (75.51%)	
**Yes**	444.00 (22.02%)	84.00 (16.50%)	111.00 (22.20%)	130.00 (24.95%)	119.00 (24.49%)	
**DM**,** n (p%)**						0.002
**No**	1,383.00 (68.60%)	370.00 (72.69%)	354.00 (70.80%)	358.00 (68.71%)	301.00 (61.93%)	
**Yes**	633.00 (31.40%)	139.00 (27.31%)	146.00 (29.20%)	163.00 (31.29%)	185.00 (38.07%)	
**COPD**,** n (p%)**						0.791
**No**	1,735.00 (86.06%)	439.00 (86.25%)	435.00 (87.00%)	442.00 (84.84%)	419.00 (86.21%)	
**Yes**	281.00 (13.94%)	70.00 (13.75%)	65.00 (13.00%)	79.00 (15.16%)	67.00 (13.79%)	
**Diagnosis**,** n (p%)**						< 0.001
**Acute and subacute liver failure**	1,181.00 (58.58%)	255.00 (50.10%)	262.00 (52.40%)	313.00 (60.08%)	351.00 (72.22%)	
**Chronic liver failure**	34.00 (1.69%)	12.00 (2.36%)	16.00 (3.20%)	5.00 (0.96%)	1.00 (0.21%)	
**Alcoholic liver failure**	284.00 (14.09%)	90.00 (17.68%)	68.00 (13.60%)	75.00 (14.40%)	51.00 (10.49%)	
**Unclassified liver failure**	517.00 (25.64%)	152.00 (29.86%)	154.00 (30.80%)	128.00 (24.57%)	83.00 (17.08%)	
**Therapy**						
**CRRT**,** n (p%)**						< 0.001
**No**	1,470.00 (72.92%)	432.00 (84.87%)	399.00 (79.80%)	368.00 (70.63%)	271.00 (55.76%)	
**Yes**	546.00 (27.08%)	77.00 (15.13%)	101.00 (20.20%)	153.00 (29.37%)	215.00 (44.24%)	
**MV**,** n (p%)**						0.001
**No**	326.00 (16.17%)	102.00 (20.04%)	94.00 (18.80%)	68.00 (13.05%)	62.00 (12.76%)	
**Yes**	1,690.00 (83.83%)	407.00 (79.96%)	406.00 (81.20%)	453.00 (86.95%)	424.00 (87.24%)	
**VP**,** n (p%)**						< 0.001
**No**	418.00 (20.73%)	139.00 (27.31%)	118.00 (23.60%)	97.00 (18.62%)	64.00 (13.17%)	
**Yes**	1,598.00 (79.27%)	370.00 (72.69%)	382.00 (76.40%)	424.00 (81.38%)	422.00 (86.83%)	
**30-day mortality**,** n(%)**						< 0.001
**No**	1,467.00 (72.77%)	428.00 (84.09%)	377.00 (75.40%)	361.00 (69.29%)	301.00 (61.93%)	
**Yes**	549.00 (27.23%)	81.00 (15.91%)	123.00 (24.60%)	160.00 (30.71%)	185.00 (38.07%)	
**90-day mortality**,** n(%)**						< 0.001
**No**	1,411.00 (69.99%)	411.00 (80.75%)	367.00 (73.40%)	341.00 (65.45%)	292.00 (60.08%)	
**Yes**	605.00 (30.01%)	98.00 (19.25%)	133.00 (26.60%)	180.00 (34.55%)	194.00 (39.92%)	

Table 2 presents the baseline features of 90-day survival and mortality groups. The median ACAG value across the study population was 20.00. The median ACAG value in non-survivors was significantly higher than that in survivors (22.00 vs. 19.25, *p* < 0.001), suggesting that ACAG is a significant prognostic marker for mortality risk. Compared with the survival group, the mortality group was heavier (*p* < 0.001), older (*p* = 0.007), had a faster heart rate (*p* = 0.003), poorer oxygen saturation (*p* = 0.016), and higher SOFA scores and MELD scores (both *p* < 0.001). Laboratory findings also suggested higher WBC and RDW (*p* < 0.001, *p* = 0.004) and longer PT and APTT (*p* < 0.001) in the deceased group. They also required more treatment with CRRT, mechanical ventilation, and vasopressors (*p* < 0.001). However, a significantly larger fraction of surviving patients had diabetes relative to non-survivors (34.09% vs. 25.12%, *p* < 0.001).

**Table 2:** Baseline characteristics of the 90-Day survival and 90-Day death Groups.

Variables	Overall (*N* = 2,016)	90d-survivor(*n* = 1,411)	90d-mortality (*n* = 605)	*p*
**Age (year)**	61.00 (52.00–71.00)	61.00 (51.00–70.00)	63.00 (52.00–73.00)	0.007
**Weight (kg)**	83.00 (68.80–98.80)	82.00 (68.10–97.40)	85.50 (70.55–102.28)	< 0.001
**Gender**,** n(%)**				0.203
**Female**	797.00 (39.53%)	545.00 (38.63%)	252.00 (41.65%)	
**Male**	1,219.00 (60.47%)	866.00 (61.37%)	353.00 (58.35%)	
**Marital status**,** n(%)**				0.318
**Divorced**	199.00 (9.87%)	137.00 (9.71%)	62.00 (10.25%)	
**Married**	964.00 (47.82%)	658.00 (46.63%)	306.00 (50.58%)	
**Single**	725.00 (35.96%)	524.00 (37.14%)	201.00 (33.22%)	
**Widowed**	128.00 (6.35%)	92.00 (6.52%)	36.00 (5.95%)	
**Race**,** n (p%)**				< 0.001
**Black**	197.00 (9.77%)	158.00 (11.20%)	39.00 (6.45%)	
**Other**	636.00 (31.55%)	384.00 (27.21%)	252.00 (41.65%)	
**White**	1,183.00 (58.68%)	869.00 (61.59%)	314.00 (51.90%)	
**Vital signs**				
**HR(beats/min)**	93.00 (80.00–109.00)	92.00 (78.00–108.00)	96.00 (82.00–110.00)	0.003
**RR (beats/min)**	20.00 (16.00–25.00)	20.00 (16.00–24.00)	21.00 (17.00–25.00)	0.059
**SpO2**	97.00 (94.00–100.00)	98.00 (95.00–100.00)	97.00 (93.00–100.00)	0.016
**Score**				
**SOFA**	10.00 (7.00–12.00)	9.00 (6.00–11.00)	11.00 (9.00–14.00)	< 0.001
**GCS**	15.00 (13.00–15.00)	15.00 (13.00–15.00)	15.00 (13.00–15.00)	0.084
**Charlson index**	6.00 (4.00–8.00)	6.00 (4.00–8.00)	6.00 (4.00–8.00)	0.571
**MELD**	18.03 (11.16–25.68)	16.78 (10.07–24.09)	21.61 (14.08–29.59)	< 0.001
**Laboratory tests**				
**ACAG**	20.00 (16.50–24.25)	19.25 (16.00–23.25)	22.00 (18.00–26.00)	< 0.001
**RBC (K/uL)**	3.03 (2.52–3.77)	3.04 (2.56–3.73)	3.02 (2.43–3.82)	0.603
**WBC (K/uL)**	11.80 (7.55–18.00)	11.10 (7.00–16.60)	14.20 (9.10–20.60)	< 0.001
**Hemoglobin (g/L)**	3.03 (2.52–3.77)	3.04 (2.56–3.73)	3.02 (2.43–3.82)	0.603
**Platelets (K/uL)**	125.00 (71.00–198.00)	121.00 (69.00–193.00)	133.00 (73.00–208.00)	0.083
**Hematocrit (%)**	28.60 (24.00–34.60)	28.60 (24.20–34.20)	28.70 (23.80–35.50)	0.096
**RDW (%)**	16.70 (14.70–19.00)	16.60 (14.70–18.60)	16.90 (14.70–19.90)	0.004
**Potassium (mEq/L)**	4.30 (3.80–5.00)	4.30 (3.80–4.90)	4.40 (3.70–5.10)	0.688
**Sodium (mEq/L)**	137.00 (133.00–141.00)	137.00 (133.00–141.00)	137.00 (132.00–141.00)	0.556
**Calcium(mEq/L)**	8.30 (7.60–8.90)	8.30 (7.70–8.80)	8.30 (7.60–8.90)	0.881
**Glucose (mg/dL)**	133.00 (105.00–185.00)	133.00 (106.00–184.00)	134.00 (102.00–187.00)	0.322
**PT (s)**	19.50 (15.50–25.80)	18.80 (15.20–24.30)	22.20 (16.10–29.60)	< 0.001
**APTT (s)**	36.40 (30.70–46.90)	35.50 (30.30–43.80)	40.50 (31.70–54.70)	< 0.001
**ALT(IU/L)**	58.00 (25.00–297.50)	54.00 (24.00–306.00)	70.00 (29.00–287.00)	0.799
**AST(IU/L)**	121.00 (52.00–488.00)	107.00 (50.00–452.00)	156.00 (64.00–575.00)	0.162
**Comorbidities**				
**Hypertension**,** n (p%)**				0.208
**No**	1,507.00 (74.75%)	1,066.00 (75.55%)	441.00 (72.89%)	
**Yes**	509.00 (25.25%)	345.00 (24.45%)	164.00 (27.11%)	
**CKD**,** n (p%)**				0.187
**No**	1,572.00 (77.98%)	1,089.00 (77.18%)	483.00 (79.83%)	
**Yes**	444.00 (22.02%)	322.00 (22.82%)	122.00 (20.17%)	
**DM**,** n (p%)**				< 0.001
**No**	1,383.00 (68.60%)	930.00 (65.91%)	453.00 (74.88%)	
**Yes**	633.00 (31.40%)	481.00 (34.09%)	152.00 (25.12%)	
**COPD**,** n (p%)**				0.349
**No**	1,735.00 (86.06%)	1,221.00 (86.53%)	514.00 (84.96%)	
**Yes**	281.00 (13.94%)	190.00 (13.47%)	91.00 (15.04%)	
**Diagnosis**,** n (p%)**				< 0.001
**Acute and subacute liver failure**	1,181.00 (58.58%)	785.00 (55.63%)	396.00 (65.45%)	
**Chronic liver failure**	34.00 (1.69%)	34.00 (2.41%)	0.00 (0.00%)	
**Alcoholic liver failure**	284.00 (14.09%)	203.00 (14.39%)	81.00 (13.39%)	
**Unclassified liver failure**	517.00 (25.64%)	389.00 (27.57%)	128.00 (21.16%)	
**Therapy**				
**CRRT**,** n (p%)**				< 0.001
**No**	1,470.00 (72.92%)	1,143.00 (81.01%)	327.00 (54.05%)	
**Yes**	546.00 (27.08%)	268.00 (18.99%)	278.00 (45.95%)	
**MV**,** n (p%)**				< 0.001
**No**	326.00 (16.17%)	263.00 (18.64%)	63.00 (10.41%)	
**Yes**	1,690.00 (83.83%)	1,148.00 (81.36%)	542.00 (89.59%)	
**VP**,** n (p%)**				< 0.001
**No**	418.00 (20.73%)	373.00 (26.44%)	45.00 (7.44%)	
**Yes**	1,598.00 (79.27%)	1,038.00 (73.56%)	560.00 (92.56%)	

### Mortality in various ACAG groups

**Fig. 1:**
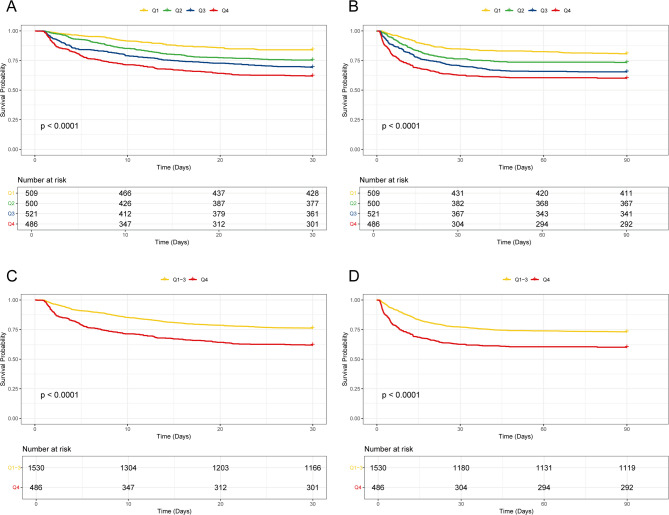
Kaplan-Meier survival curves for all-cause mortality in ACAG quartiles. (**A**) 30-day and (**B**) 90-day mortality: showing stratification of ACAG quartiles (Q1 to Q4), showing a progressive decrease in survival from the lowest to the highest quartile (*p* < 0.0001). (**C**) 30-day and (**D**) 90-day mortality, pooled groups: comparing the highest quartile (Q4) to the combined quartiles (Q1 ~ 3) highlights a significant reduction in survival from Q4 (*p* < 0.0001).

By ACAG level in groups 1–4, the 30-day mortality was 15.91%, 24.60%, 30.71%, and 38.07%, while the 90-day mortality was 19.25%, 26.60%, 34.55%, and 39.92%. It was revealed that the mortality increased with the ACAG levels. The Kaplan-Meier survival curves also demonstrated this relationship. (Fig. 1).

### Relationship between all-cause mortality and ACAG

Cox regression modeling results showed a correlation between higher ACAG and higher 30- and 90-day mortality risk (Table 3). In Model 1, the HR for 30-day and 90-day mortality were 1.062 (1.050–1.074) and 1.059 (1.047–1.070) (both *P* < 0.001). In Model 3, the HR decreased to 1.032 (1.019–1.046) and 1.031 (1.018–1.045) (both *P* < 0.001). From Model 1 to Model 3, HR decreased slightly, but remained greater than 1, indicating that elevated ACAG was significantly and positively associated with 30- and 90-day mortality in patients with liver failure. The hazard ratios for all quartiles (Q2–Q4) in each model were significantly higher than that of the reference group (Q1), showing a clear dose-response relationship and confirming the predictive capability of ACAG for 30- and 90-day mortality. All variables had VIF < 3, supporting stable model estimation (Table S3).

**Table 3:** Cox proportional risk ratio (HR) for all-cause mortality.

Categories	Model 1			Model 2			Model 3		
	HR (95% CI)	*P* value	*P* for trend	HR (95% CI)	*P* value	*P* for trend	HR (95% CI)	*P* value	*P* for trend
30-day mortality									
**Continuous**	1.062(1.050–1.074)	< 0.001		1.060(1.048–1.072)	< 0.001		1.032(1.019–1.046)	< 0.001	
**Quartile**			< 0.001			< 0.001			0.001
**Q1**	1.00 (Reference)			1.00 (Reference)			1.00 (Reference)		
**Q2**	1.628 (1.230–2.155)	0.001		1.596 (1.205–2.114)	0.001		1.432 (1.078–1.901)	0.013	
**Q3**	2.162 (1.655–2.825)	< 0.001		2.118 (1.619–2.772)	< 0.001		1.516 (1.145–2.006)	0.004	
**Q4**	2.893 (2.228–3.757)	< 0.001		2.757 (2.117–3.589)	< 0.001		1.706 (1.281–2.273)	< 0.001	
**90-day mortality**									
**Continuous**	1.059 (1.047–1.070)	< 0.001		1.057 (1.046–1.069)	< 0.001		1.031 (1.018–1.045)	< 0.001	
**Quartile**			< 0.001			< 0.001			0.001
**Q1**	1.00 (Reference)			1.00 (Reference)			1.00 (Reference)		
**Q2**	1.466 (1.130–1.904)	0.004		1.440 (1.109–1.870)	0.006		1.316 (1.011–1.714)	0.041	
**Q3**	2.018 (1.578–2.581)	< 0.001		1.982 (1.547–2.539)	< 0.001		1.470 (1.135–1.904)	0.004	
**Q4**	2.561 (2.009–3.266)	< 0.001		2.453 (1.918–3.135)	< 0.001		1.583 (1.210–2.069)	0.001	

Model 1:unadjusted. Model 2:adjusted for age, weight, HR. Model 3: adjusted for age, weight, HR, SpO2, SOFA, MELD, WBC, RDW, PT, APTT, DM, CRRT, MV, VP.

**Fig. 2:**
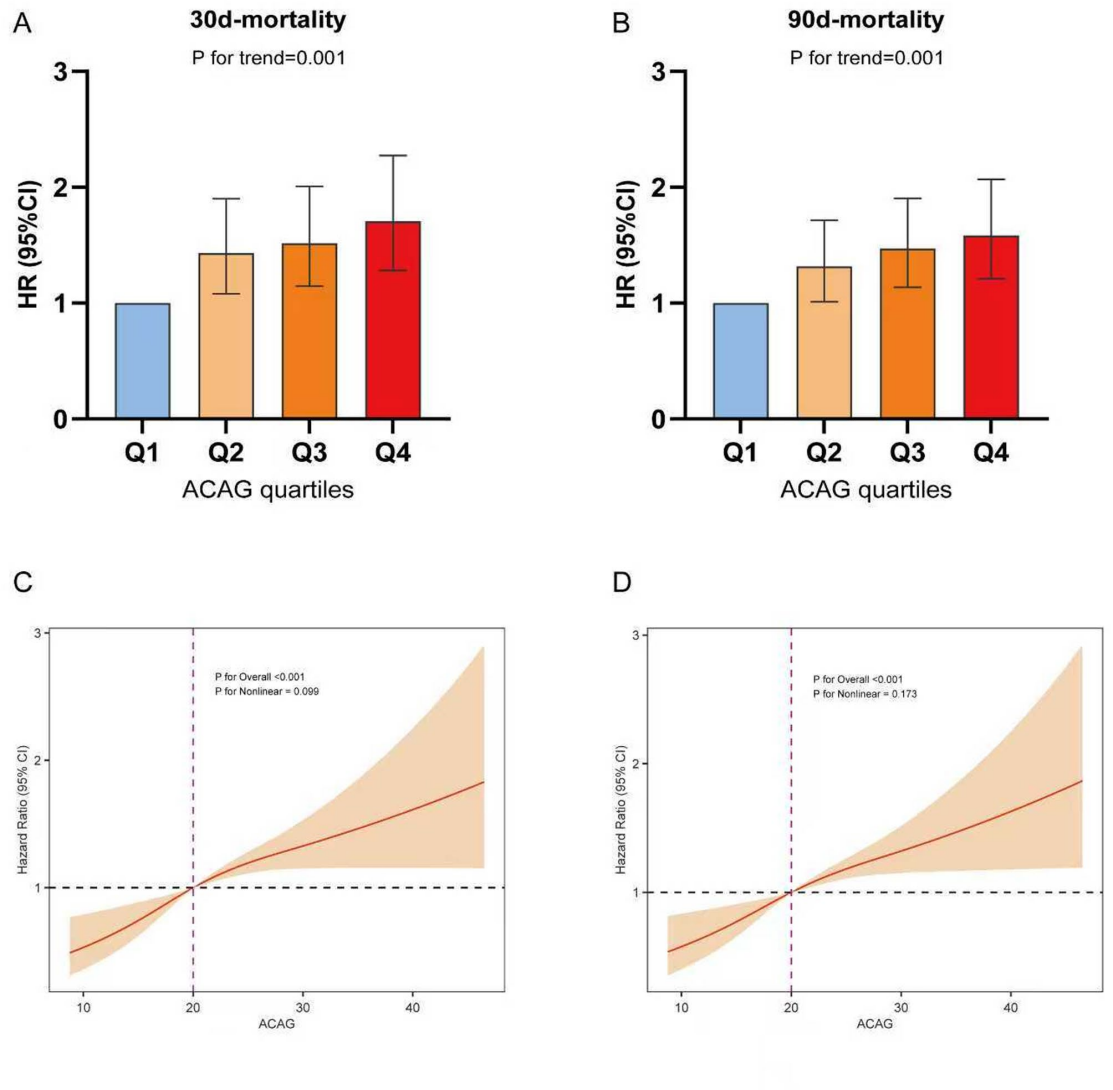
HR (95% CI) for all-cause mortality by ACAG quartiles after correction for AGE, WEIGHT, HR, SpO2, SOFA, MELD, WBC, RDW, PT, APTT, TDM, CRRT, MV, VP. Where Q1 is the reference value. (**A**) HR (95% CI) for 30-day mortality by ACAG quartile. (**B**) HR (95% CI) for 90-day mortality by ACAG quartile. (**C**) RCS curves for 30-day and (**D**) 90-day mortality.

The RCS showed that elevated ACAG levels were related to 30- and 90- day mortality (*p* < 0.001), and none of the nonlinear tests reached the threshold of significance (*p* = 0.099, *p* = 0.173) (Fig. 2).

### Comparative predictive value of ACAG and MELD in patients with liver failure

**Fig. 3:**
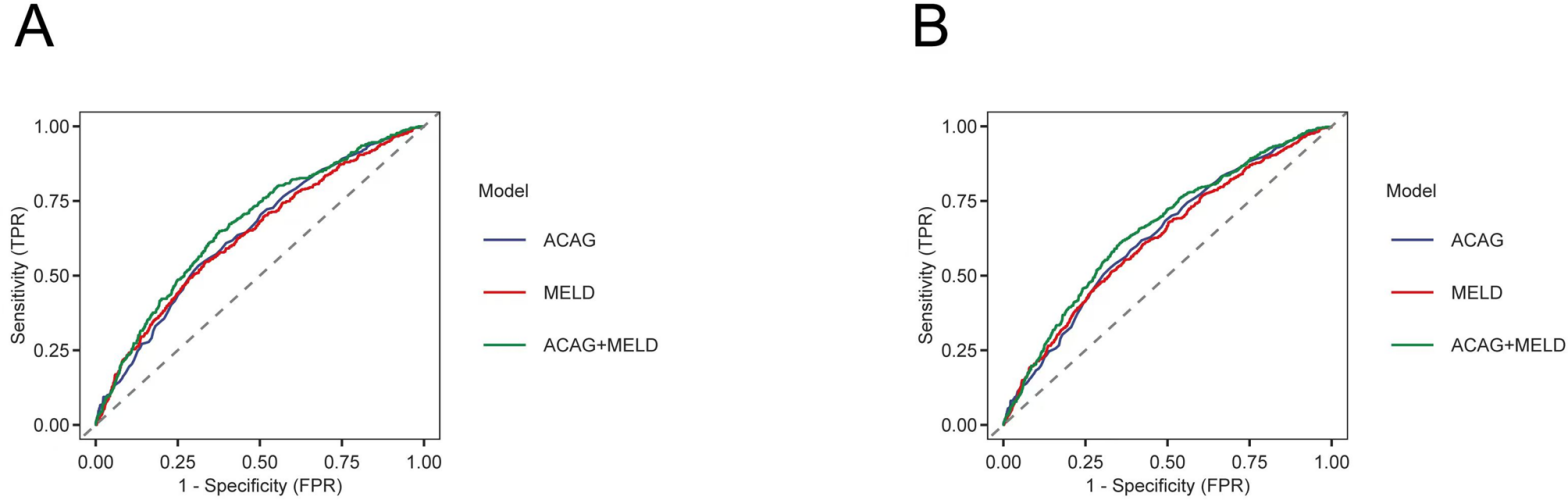
ROC curves for predicting all-cause mortality. ROC curves for ACAG, MELD, and combined prediction of 30-day (**A**) and 90-day (**B**) mortality.

ACAG demonstrates high predictive value for both 30- and 90-day mortality. For 30-day mortality, the AUC was 0.641 (0.612–0.669) for ACAG alone, 0.634 (0.605–0.663) for MELD alone, and 0.668 (0.640–0.696) for ACAG in combination with MELD. The AUC of ACAG compared with MELD showed no statistically significant difference (Z = 0.381, *P* = 0.7035), and both were lower than the AUC of their combination (Z = − 3.317, *P* = 0.0009 and Z = − 3.566, *P* = 0.0004). For 90-day mortality, the AUC was 0.630 (0.604–0.657) for ACAG alone, 0.621 (0.594–0.648) for MELD alone, and 0.654 (0.630–0.680) for ACAG combined with MELD. There was no statistically significant difference in AUC between ACAG and MELD (Z = 0.615, *P* = 0.5387). The AUC of the combined model was higher than that of either model alone (Z = − 3.151, *P* = 0.0016 and Z= − 3.670, *P* = 0.0002). (Table 4; Fig. 3)

**Table 4:** Prognostic accuracy of ACAG and MELD.

Prognostic marker	Specificity	Sensitivity	NPV	PPV	Accuracy	AUC(95%CI)
30-day mortality						
**ACAG**	0.686	0.533	0.837	0.278	0.652	0.641 (0.612–0.669)
**MELD**	0.665	0.547	0.836	0.319	0.639	0.634 (0.605–0.663)
**ACAG+MELD**	0.624	0.649	0.861	0.331	0.629	0.668 (0.640–0.696)
**90-day mortality**						
**ACAG**	0.674	0.527	0.779	0.395	0.631	0.630 (0.604–0.657)
**MELD**	0.670	0.515	0.773	0.386	0.625	0.621 (0.594–0.648)
**ACAG+MELD**	0.645	0.542	0.803	0.410	0.635	0.654 (0.630–0.680)

Abbreviations: HR, heart rate; RR, respiratory rate; SpO2, blood oxygen saturation; SOFA, sepsis-associated organ failure assessment score; GCS, Glasgow Coma Score; MELD, model for end-stage liver disease; RBC, red blood cell; WBC, white blood cell; RDW, red blood cell distribution width; PT, prothrombin time; APTT, activated partial thromboplastin time; ALT, alanine aminotransferase; AST, aspartate aminotransferase; CKD, chronic kidney disease; DM, diabetes mellitus; COPD, chronic obstructive pulmonary disease; CRRT, continuous renal replacement therapy; MV, mechanical ventilation; VP, vasopressin; NPV, negative predictive value; PPV, positive predictive value; area under the AUC curve.

### Subgroup analysis

**Fig. 4:**
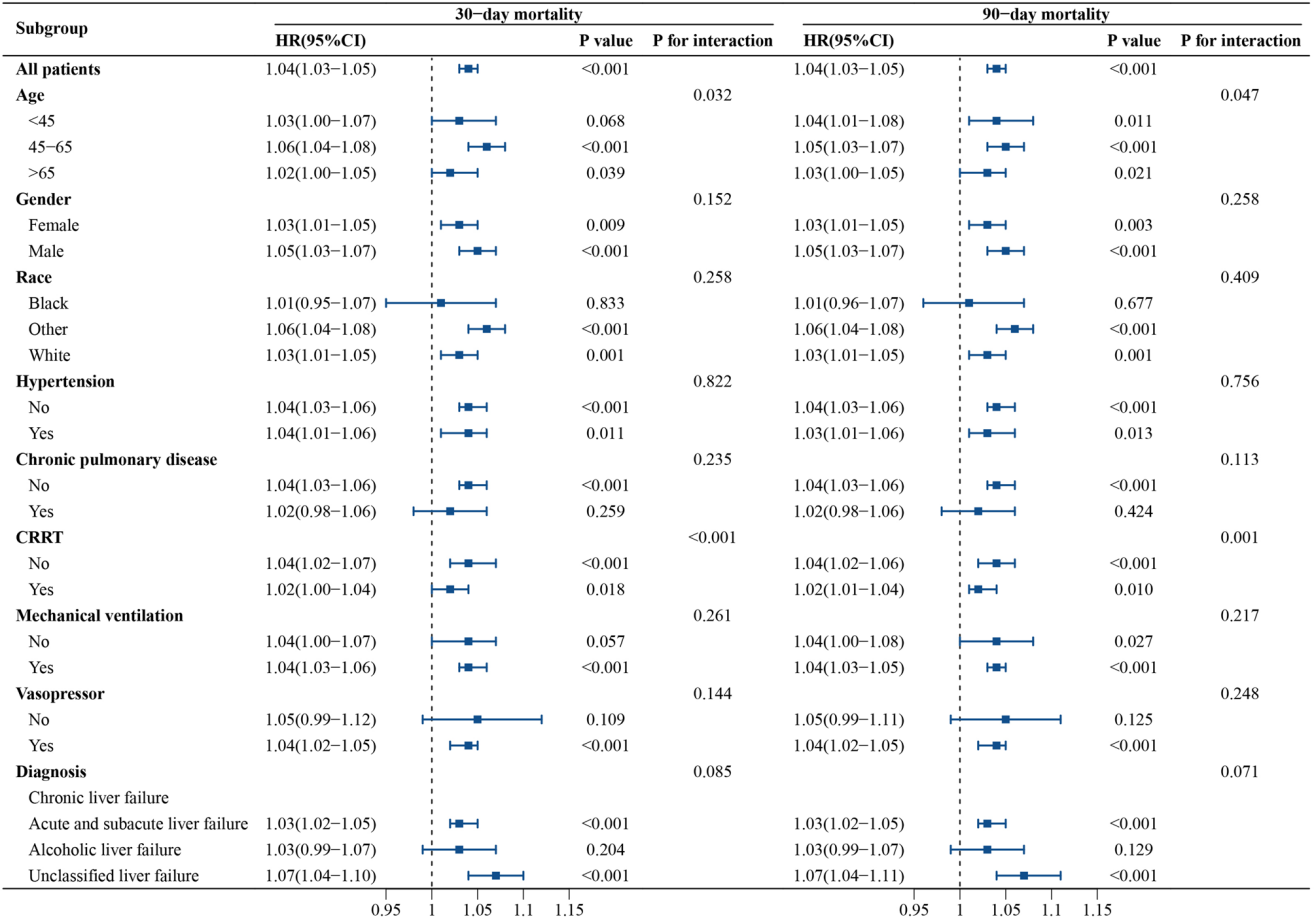
Forest plot of HR for 30-day and 90-day mortality in different subgroups. HR was adjusted according to age, weight, HR, SpO2, SOFA, MELD, WBC, RDW, PT, APTT, TDM, CRRT, MV, VP.

Subgroup analysis of ACAG based on demographic and clinical characteristics revealed a significant association between elevated ACAG levels and increased 30- and 90-day mortality in patients with liver failure. Nevertheless, significant interactions were observed in age (*p* = 0.032 and 0.047) and CRRT subgroups (*p* < 0.001 and 0.001) for 30- and 90-day mortality, suggesting that there were variations in mortality risk prediction tied to age and CRRT (Fig. 4). Subgroup analysis showed non-survivors had significantly higher median ACAG than survivors across all liver failure subtypes (Table S4). The divergence was most pronounced among patients with acute and subacute liver failure, in whom the median ACAG rose from 20.25 (16.50–24.75) in survivors to 23.00 (18.88–27.25) in non-survivors for 30- day, and from 20.25 (16.50–24.75) to 22.75 (18.63–27.00) for 90- day (both *P* < 0.001). Similar, albeit smaller, inter-group differences were observed for alcoholic and unclassified liver failure, and remained statistically significant throughout Fig. 4.

## Discussion

In this MIMIC-IV retrospective cohort of 2,016 liver-failure patients, 30- and 90-day mortality rose stepwise from Q1 to Q4. Comparing survival between the highest ACAG quartile (Q4) and the combined group of the three lower quartiles (Q1–Q3) simplifies risk stratification into high risk versus non-high risk. This approach is more intuitive for rapid bedside assessment or designing interventions specifically targeting the highest-risk cohort. Following multivariate Cox regression adjustment, HRs remained elevated across all quartiles (Q2-Q4) versus Q1, albeit slightly attenuated. ACAG showed a strong, dose-dependent association with 30- and 90-day mortality, both as a continuous and as a categorical variable; RCS curves confirmed linearity. The 90-day mortality group exhibited significantly higher ACAG levels than survivors, confirming a positive correlation between ACAG and short-term mortality in patients with liver failure and supporting its value as a prognostic marker. The AUC indicated that ACAG exhibited predictive performance comparable to that of MELD for 30- and 90-day mortality. The combination of ACAG and MELD further improved sensitivity and negative predictive value (NPV), outperforming each model individually. These findings indicated that ACAG retains robust predictive value and complemented the MELD score. Their combined use improved risk stratification, facilitating the early identification of high-risk patients and enabling timely intervention to slow disease progression in individuals with liver failure.

The results of subgroup analyses showed that ACAG served as a stable and reliable prognostic indicator of liver failure for forecasting 30- and 90-day mortality across different populations and clinical scenarios. The ACAG level was significantly higher in non-survivors across all subgroups. This difference was highly significant (*P* < 0.001) in the total cohort and in the acute/subacute and unclassified subgroups, while it was statistically significant in the alcoholic liver failure subgroup (*P* = 0.029 and 0.033 for 30- and 90-day mortality, respectively). However, significant interactions between 30- and 90-day mortality were observed in the age (*p* = 0.032, *p* = 0.047) and CRRT-treated subgroup (*p* < 0.001, *p* = 0.001). Many studies have established the consistent predictive value of ACAG for mortality across different age groups in multiple diseases^18,22–24^. However, our study found that individuals aged 45–65 years and those not receiving CRRT showed a numerically higher hazard ratio at both 30- and 90- day mortality. These observations might reflect chance, residual confounding, selection bias (e.g., CRRT indication), or differential care patterns, and require further investigation.

Beyond the critical roles of the respiratory and renal systems, the liver also plays a crucial role in acid base homeostasis through lactate and Cori cycle metabolism, albumin synthesis, ketone-body generation, and urea cycle activity. Moreover, extrahepatic complications such as hepatic encephalopathy, ascites, and acute renal failure disturb acid–base balance, thereby rendering hepatic dysfunction a complex acid–base imbalance^25–28^. Serum AG, a key indicator of acid–base status, is widely used to diagnose and monitor acid–base disorders and to predict poor prognosis across multiple diseases^29–32^. Albumin, the predominant plasma protein, maintains colloid osmotic pressure, acts as an antioxidant and anti-inflammatory agent, modulates immunity, transports ligands, and mediates signaling, thereby preserving physiological homeostasis. Its concentration reflects nutritional state, hepatic synthesis, and endothelial integrity^33–35^. In liver failure, impaired albumin concentration and function reflect disease severity^33^. Hypoalbuminaemia reduces the accuracy of AG in diagnosing the degree of acid-base imbalance. In advanced liver disease, hypoalbuminaemia usually results from decreased albumin synthesis attributable to hepatocyte depletion and the suppressive effects of systemic inflammation^36^. Metabolic acidosis, hypoproteinemia, inflammation, and oxidative stress often coexist in patients with end-stage liver disease^37^; relying on either AG or albumin alone may underestimate disease severity. We therefore used ACAG—a more accurate composite indicator—to predict short-term prognosis in liver failure.

Up to now, we have found only 3 studies that have explored the relationship between ACAG and liver disease. In the first study, Lu et al. demonstrated the significant prognostic value of ACAG for NAFLD, particularly in overweight/obese populations. A predictive model incorporating ACAG, waist circumference, and ALT achieved strong performance (AUC = 0.834)^38^. In the second study, Bai et al. confirmed the clinical utility of ACAG for identifying NAFLD risk, demonstrating population-specific efficacy particularly in individuals under 60 years. They identified a nonlinear, inverted U-shaped association between ACAG and NAFLD (inflection point: 23.05 mmol/L), indicating a bidirectional risk relationship mediated by ACAG levels^39^. In the third study, Pan et al. examined the association between ACAG and clinical outcomes in individuals suffering from cirrhotic sepsis for the first time, confirming that higher ACAG levels were independently correlated with increased 28-day mortality^14^. Furthermore, the study by Yu et al. was the first to systematically evaluate the association between in-hospital mortality and AG in patients with acute liver failure; they found that AG > 20 mmol/L was independently associated with mortality and provided predictive performance (AUC = 0.666) comparable to that of the MELD score. The authors concluded that hypoalbuminemia caused by liver failure exerts only a minor effect on AG, so AG alone remains adequate for assessing metabolic acidosis^40^. Although previous studies had highlighted the prognostic value of ACAG in liver disease, none had specifically examined its association with short-term outcomes of liver failure. Our findings demonstrated the role and predictive value of ACAG in liver failure.

Multiple mechanisms account for the correlation between ACAG and liver failure^41,42^. First, liver failure increases AG via lactate accumulation, ketogenesis, or renal insufficiency, reflecting tissue hypoxia and global metabolic derangement. Second, concomitant hypoalbuminaemia lowers AG and compromises its reliability as an indicator of acid-base status; albumin is also an established inflammatory and nutritional biomarker that predicts outcome. Third, elevated ACAG closely mirrors systemic inflammation and oxidative stress—drivers of hepatocellular injury and multi-organ failure. Because ACAG adjusts for albumin, it minimises diagnostic error; its elevation thus identifies severe acid-base imbalance that is mechanistically linked to mortality^43^. To enhance prognostic accuracy in liver failure patients, our study incorporated ACAG as a supplementary measure alongside the MELD score.

Our study has the following strengths: (1) the large sample size provides strong statistical power to validate the prognostic value of ACAG; (2) we used ACAG instead of AG to correct for albumin-induced underestimation of acidosis in hypoproteinaemic patients, thereby reducing false-negative results; (3) precise stratification by ACAG quartiles, age strata, multivariable adjustment, and subgroup analyses confirms the robustness of ACAG; (4) integration of ACAG with MELD improves 30- and 90-day mortality prediction.

Of course, our study has some limitations. First, retrospective studies are inevitably affected by data completeness and confounders. Although we adjusted for confounders through multivariate analyses, there may still be potential confounders that were not considered, resulting in bias. Second, our findings require external validation in geographically and ethnically diverse populations, including non-ICU settings and transplant centers, to assess broader applicability. Finally, the etiology of liver failure could not be traced due to the limitations of the database to establish causality, and it could not be ruled out that liver failure was secondary to other organ failure. Furthermore, the primary etiology of hepatic failure varies among different countries and regions, so it is uncertain whether the conclusions of this study apply to other countries and races. Future prospective, multicenter studies—ideally comparing the prognostic performance of ACAG across different critical illnesses—are warranted to define its disease-specific utility and consolidate the present results.

## Conclusion

These findings revealed a strong, direct and linear association between ACAG and both 30- and 90-day mortality in patients with liver failure. The discriminative ability of ACAG was comparable to that of MELD, and the two variables together augmented prognostic accuracy. ACAG thus provides an inexpensive yet highly sensitive adjunct for risk stratification, facilitating early identification of patients destined to progress to severe liver failure and informing clinical decision-making. Nevertheless, large-scale, multicenter, prospective studies are essential to determine the universal applicability and unbiased prognostic value of ACAG in patients with liver failure.

## Supplementary Information

Below is the link to the electronic supplementary material.

Supplementary Material 1

## Data Availability

The data for this study were extracted from the MIMIC-IV database at https://mimic-iv.mit.edu/.
