# Supplementary material for: Association of ACAG with short-term mortality in liver failure patients: a retrospective analysis based on the MIMIC-IV database
Source: Sci Rep. 2026 Mar 21;16:14482. doi: 10.1038/s41598-026-39253-5 (PMC13149636; doi:10.1038/s41598-026-39253-5)
Supplement: Supplementary file 1 — Supplementary Material 1 [file 41598_2026_39253_MOESM1_ESM.pdf]

Table S1 The missing number of variables.

Variable Names	level	Overall	Missing
n		2016	
Age(year)		61 (19-98)	0
Weight(kg)		83.045 (30.7-230.25)	1
HR (beats/min)		93 (0-182)	0
RR (beats/min)		20 (0-54)	0
SpO2		97 (20-963)	0
Charlson index		6 (0-17)	0
GCS		15 (13-15)	0
SOFA		10 (0-22)	0
MELD		18.029 (-10.738-60.28)	0
Hemoglobin(g/L)		9.3 (3.6-19.8)	0.6
Platelets(K/uL)		124 (5-1647)	0.7
RDW (%)		16.6 (11.6-36)	1.2
WBC(K/uL)		11.8 (0.2-269.8)	0.5
RBC (K/uL)		3.03 (1.07-6.99)	0.5
Glucose(mg/dL)		133 (15-2044)	0
Potassium(mEq/L)		4.3 (2.1-9.3)	0.1
Sodium(mEq/L)		137 (100-169)	0
Calcium(mEq/L)		8.3 (4.2-18.9)	0
PT(s)		19.5 (9.6-150)	1.6
APTT(s)		36.4 (19.8-150)	1.7
AST(IU/L)		121 (5-28275)	0.1
ALT(IU/L)		58 (5-13330)	0.9
Hematocrit (%)		28.6 (11.5-64.6)	0.4
Gender, n(%)	F	797 (39.53)	0
Gender, n(%)	M	1219 (60.47)	
Marital status, n(%)	DIVORCED	170 (10.19)	17.2
Marital status, n(%)	MARRIED	790 (47.33)	
Marital status, n(%)	SINGLE	602 (36.07)	
Marital status, n(%)	WIDOWED	107 (6.41)	
Race, n(%)	BLACK	197 (9.77)	0
Race, n(%)	Other	636 (31.55)	

**Race, n(%)**	WHITE	1183 (58.68)	
**Chronic pulmonary disease, n(%)**	No	1735 (86.06)	0
**Chronic pulmonary disease, n(%)**	Yes	281 (13.94)	
**Chronic kidney disease, n(%)**	No	1572 (77.98)	0
**Chronic kidney disease, n(%)**	Yes	444 (22.02)	
**Diabetes mellitus, n(%)**	No	1383 (68.60)	0
**Diabetes mellitus, n(%)**	Yes	633 (31.40)	
**Hypertension, n(%)**	No	1507 (74.75)	0
**Hypertension, n(%)**	Yes	509 (25.25)	
**CRRT, n(%)**	No	1470 (72.92)	0
**CRRT, n(%)**	Yes	546 (27.08)	
**Mechanical ventilation, n(%)**	No	326 (16.17)	0
**Mechanical ventilation, n(%)**	Yes	1690 (83.83)	
**Vasopressor, n(%)**	No	418 (20.73)	0
**Vasopressor, n(%)**	Yes	1598 (79.27)	
**Acute and subacute liver failure**		1181 (58.58)	0
**Chronic liver failure**		34 (1.69)	
**Alcoholic liver failure**		284 (14.09)	
**Unclassified liver failure**		517 (25.64)	

Abbreviations: HR, heart rate; RR, respiratory rate; SpO2, blood oxygen saturation; GCS, Glasgow Coma Scale; SOFA, Sepsis-Related Organ Failure Assessment Score; MELD, Model for End-Stage Liver Disease; RDW, red blood cell distribution width; WBC, white blood cells; RBC, Red Blood Cells; PT, prothrombin time; APTT, Activated partial thromboplastin time; AST, Aspartate aminotransferase; ALT, Alanine aminotransferase; CRRT, Continuous Renal Replacement Therapy.

Table S2 Univariate Cox proportional analysis for 90-day mortality.

**Variables**	**HR (95%CI)**	**P**
**ACAG**	1.059（1.047-1.070）	<0.001
**Age(year)**	1.008(1.002-1.013)	0.005
**Weight (kg)**	1.006 (1.003-1.009)	<0.001
**Race, n (p%)**		
**Black**	1.00 (Reference)	
**Other**	2.303(1.643-3.227)	<0.001
**White**	1.400(1.004-1.952)	0.048
**SOFA**	1.141(1.118-1.164)	<0.001
**HR (beats/min)**	1.005(1.002-1.009)	0.003

**SpO2**	0.975(0.964-0.986)	<0.001
**RDW (%)**	1.035(1.012-1.059)	0.003
**WBC (K/uL)**	1.012(1.009-1.016)	<0.001
**PT (s)**	1.011(1.007-1.015)	<0.001
**APTT (s)**	1.007 (1.004-1.009)	<0.001
**MELD**	1.032(1.025-1.039)	<0.001
**Diabetes mellitus, n(%)**		
**No**	1.00 (Reference)	
**Yes**	0.684(0.569-0.822)	<0.001
**Mechanical ventilation, n(%)**		
**No**	1.00 (Reference)	
**Yes**	1.748(1.347-2.269)	<0.001
**Vasopressor, n(%)**		
**No**	1.00 (Reference)	
**Yes**	3.822(2.821-5.179)	<0.001
**CRRT, n(%)**		
**No**	1.00 (Reference)	
**Yes**	2.741(2.335-3.217)	<0.001
**Diagnosis, n(%)**		
**Acute and subacute hepatic failure**	1.00 (Reference)	
**Chronic hepatic failure**	0 (0-lnf)	0.985
**Alcoholic hepatic failure**	0.793(0.625-1.007)	0.058
**Hepatic failure, unspecified**	0.676(0.554-0.825)	<0.001

Abbreviations: ACAG, albumin-corrected anion gap; SOFA, Sepsis-Related Organ Failure Assessment Score; HR, heart rate; SpO2, blood oxygen saturation; RDW, red blood cell distribution width; WBC, white blood cells; PT, prothrombin time; APTT, Activated partial thromboplastin time; MELD, Model for End-Stage Liver Disease; CRRT, Continuous Renal Replacement Therapy.

Table S3: Multicollinearity analysis of covariates used in Cox model 3

**Covariate**	**VIF for 30-day mortality**		**VIF for 90-day mortality**	
	**ACAG (continuous)**	**ACAG (quartile)**	**ACAG (continuous)**	**ACAG (quartile)**
**Age**	1.1627	1.1607	1.1614	1.1606
**Weight**	1.1204	1.1215	1.1166	1.1206
**HR**	1.0884	1.0987	1.0954	1.1031
**SPO2**	1.0486	1.0532	1.0468	1.0525
**RDW**	1.1137	1.1177	1.1139	1.1172
**WBC**	1.0344	1.0378	1.0358	1.0400
**PT**	1.5682	1.5847	1.5646	1.5874
**APTT**	1.0823	1.0899	1.0814	1.0909
**ACAG**	1.2923	1.3091	1.3043	1.3114

**SOFA**	1.4930	1.4690	1.5025	1.4798
**MELD**	2.0559	2.1573	2.1012	2.2116
**DM**	1.0709	1.0625	1.0780	1.0664
**CRRT**	1.3331	1.3167	1.3357	1.3220
**MV**	1.0780	1.0797	1.0864	1.0901
**VP**	1.1227	1.1239	1.1326	1.1338

Abbreviations: HR, heart rate; SpO2, blood oxygen saturation; RDW, red blood cell distribution width; WBC, white blood cell; PT, prothrombin time; APTT, activated partial thromboplastin time; ACAG, albumin-corrected anion gap; SOFA, sepsis-associated organ failure assessment score; MELD, model for end-stage liver disease; DM, diabetes mellitus; CRRT, continuous renal replacement therapy; MV, mechanical ventilation; VP, vasopressin

Table S4: Comparison of ACAG levels according to 30- and 90-day survival across liver-failure subgroups

	**30-day outcome**		**P-value**	**90-day outcome**		**P-value**
	**Survivors**	**Non-survivors**		**Survivors**	**Non-survivors**	
Total	1,467	549		1,411	605	
ACAG[median(P25-P75)]	19.25(16.00-23.25)	22.25(18.25-26.50)	<0.001	19.25(16.00-23.25)	22.00(18.00-26.00)	<0.001
Acute and subacute liver failure	821	360		785	396	
ACAG[median(P25-P75)]	20.25(16.50-24.75)	23.00(18.88-27.25)	<0.001	20.25(16.50-24.75)	22.75(18.63-27.00)	<0.001
Alcoholic liver failure	210	74		203	81	
ACAG[median(P25-P75)]	18.88(15.50-22.00)	20.63(17.25-24.00)	0.029	19.00(15.50-22.00)	20.50(16.50-23.75)	0.033
Unclassified liver failure	402	115		389	128	
ACAG[median(P25-P75)]	18.25(165.75-21.75)	20.50(17.75-25.00)	<0.001	18.25(15.75-21.50)	20.38(17.75-24.38)	<0.001

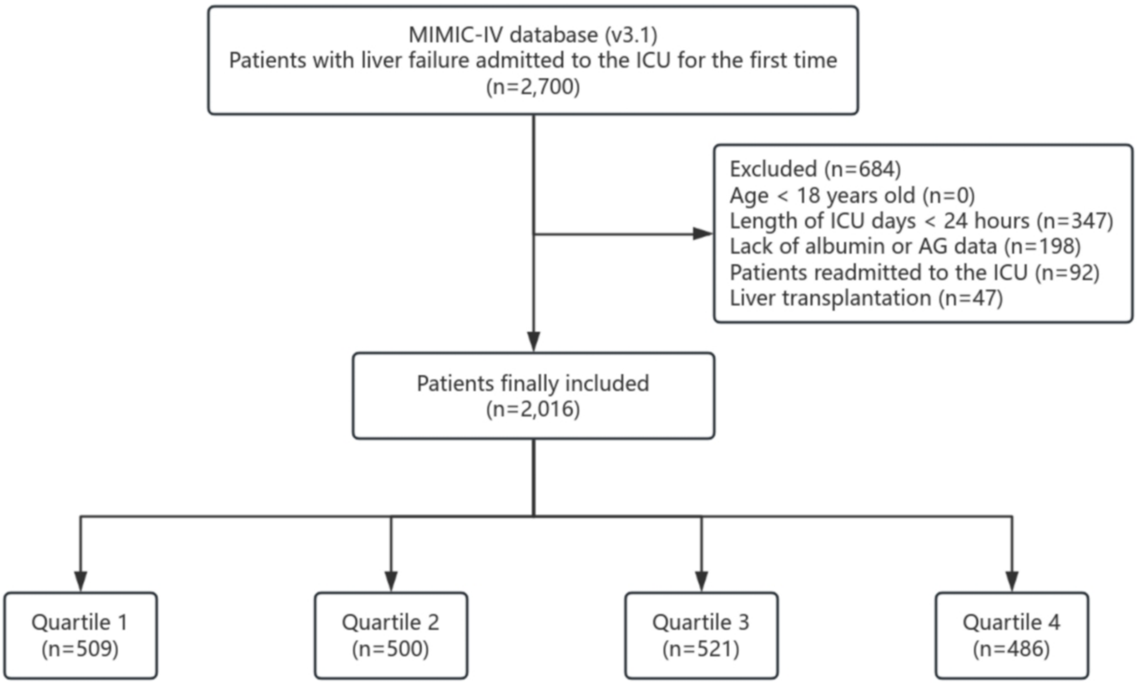

Figure S1: Flow chart. ICU, intensive care unit; AG, anion gap.
